# Traditional Korean Medicine Treatment for Patients with Wilting Disorder: A Literature Review of In Vivo Studies

**DOI:** 10.1155/2018/5601846

**Published:** 2018-11-13

**Authors:** Sung-Jin Kim, Yeon-Cheol Park, Yong-Hyeon Baek, Byung-Kwan Seo

**Affiliations:** Department of Acupuncture & Moxibustion, Kyung Hee University Hospital at Gangdong, 149 Sangil-dong, Gangdong-gu, Seoul 134-727, Republic of Korea

## Abstract

Wilting disorder is an abnormal condition characterized by weakness and paralysis of the upper and lower extremities. Pathogenesis and treatment target of the disorder are unclear; hence, allopathic treatment is generally used to relieve the symptoms. To investigate the treatment mechanism and effect of Traditional Korean Medicine (TKM) in patients with wilting disorder, we reviewed in vivo studies that focused on the effect of TKM on the main symptoms of wilting disorder and treatment of the diseases that can cause these symptoms. We electronically searched the PubMed, Cochrane, and CNKI (China National Knowledge Infrastructure) databases using the following search terms: (weakness OR motor function disorder) (myasthenia gravis OR Guillain-Barre syndrome OR amyotrophic lateral sclerosis OR paralysis OR polymyositis OR muscular dystrophy) AND (herbal medicine OR acupuncture OR bee-venom OR pharmacoacupuncture OR electro-acupuncture OR moxibustion). We selected 11 studies that demonstrated the effect of TKM treatment on the main symptoms of wilting disorder. In these studies, inducted models of amyotrophic lateral sclerosis, myasthenia gravis, Duchenne muscular atrophy, polymyositis, and Guillain-Barre syndrome were used. With regard to treatment, herbal medicine was used in five studies, and acupuncture and bee-venom pharmacoacupuncture were used in three studies each. Future research is needed to determine the effectiveness of TKM treatment in patients with diseases that can cause the main symptoms of wilting disorder.

## 1. Introduction

Wilting disorder, also called “Wei-syndrome” in Traditional Korean Medicine (TKM), is defined as an abnormal condition characterized by weakness and paralysis of the upper and lower extremities in the Korean Standard Classification of Disease and Cause of Death (KCD classification). The main symptoms of Wilting disorder are loss of muscle tone in the weakened or paralyzed parts, decrease in muscle strength, and decrease or disappearance of reflexes. In the TKM, the cause of wilting disorder is divided into the factors outside of the body and the factors inside of the body. The external factors include the traumatic events or overwork that can damage bones, joints, and soft tissues, fever causing neurologic dysfunction, and disease which have long morbidity period. The internal factors include energy reduction caused by wrong lifestyle or diseases and malfunctioning of viscera such as liver, heart, spleen, lung, and kidney [1, 2].

There have been arguments between TKM doctors about the TKM treatment of wilting disorder, but the mechanisms and treatment effects of each treatment have remained unclear, despite many modern studies on wilting disorder in TKM [1, 2].

The purpose of this study was to review clinical studies focusing on the treatment mechanisms and effects of TKM treatments on wilting disease. A modern study focusing on wilting disorder suggested that diseases such as myasthenia gravis, Guillain-Barre syndrome, amyotrophic lateral sclerosis, paralysis, polymyositis, and muscular dystrophy could manifest clinical signs similar to those of wilting disorder, such as weakness, paralysis, and loss of motion [3].

So we tried to investigate the treatment mechanisms and effects of TKM treatments in these diseases. This study aimed to investigate the research trends of TKM treatment for diseases that can cause wilting disorder through utilizing various databases and analyze the results. This process will provide information and evidences of TKM treatment to treat wilting disorder in clinical practice and designing a clinical research about TKM treatments.

## 2. Methods

### 2.1. Search Strategies

We searched the PubMed, Cochrane, and CNKI databases from inception through April 2018. No language limitation was applied. Key search terms were (myasthenia gravis OR Guillain-Barre syndrome OR amyotrophic lateral sclerosis OR paralysis OR polymyositis OR muscular dystrophy) AND (weakness OR motor function disorder) AND (herbal medicine OR acupuncture OR bee-venom OR pharmacoacupuncture OR electro-acupuncture OR moxibustion).

### 2.2. Data Extraction

The data were extracted and grouped using a data extraction form that included the animal (sex, species, and strain), the disease model type (type of disease: myasthenia gravis, Guillain-Barre syndrome, amyotrophic lateral sclerosis, paralysis, polymyositis, and muscular dystrophy), the type of intervention, and the outcome measures (electrophysiological status, histological and biochemical measurement, and motor function value index). The data were extracted primarily by one author and checked by all other authors.

## 3. Results

**Figure 1:**
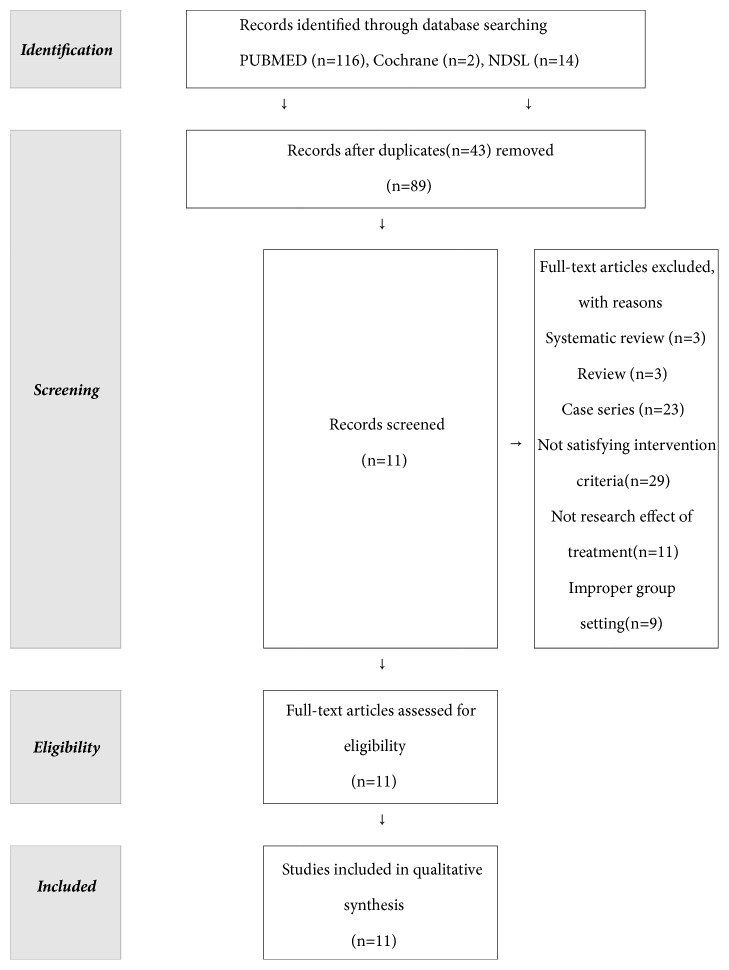
Flow diagram of study selection process.

We extracted 159 studies by using our key terms and read their titles and abstracts. Out of these 159 articles, 89 records remained after the duplicates were removed. Of these 89 articles, six articles were systematic review or review of literature, and 23 articles were case reports. There were 29 articles not satisfying intervention criteria; and 11 articles were not research studies on the treatment effect. Nine articles were excluded because of improper group-setting. After screening, we selected 11 animal model studies for analysis (Figure 1). We analyzed the animal models for wilting disorder in 11 studies (Table 1). Of the 11 studies, four studies used amyotrophic lateral sclerosis model; myasthenia gravis and multiple sclerosis-induced animal models were used in two studies each; and Duchenne muscular atrophy, polymyositis, and Guillain-Barre syndrome-induced models were used in one study each. With regard to the intervention, five studies investigated the effect of herbal medicine, and three studies each focused on acupuncture and bee-venom pharmacoacupuncture as treatment.

## 4. Discussion

Wilting disorder can occur because of the abnormality of the muscle, neuromuscular junction, peripheral nerve, and lower and upper motor neuron.

The main symptoms of wilting disorder include flaccid palsy, muscle atrophy, loss of reflex, muscular fasciculation, muscular contracture, and reaction of degeneration. Diseases that could cause this condition include myasthenia gravis, Guillain-Barre syndrome, amyotrophic lateral sclerosis, multiple sclerosis, paralysis, polymyositis, and muscular dystrophy.

Myasthenia gravis is an autoimmune disease that results from abnormality of the neuromuscular junction. The pathogenesis involves the action of antibodies to block or destroy nicotinic acetylcholine receptors at the neuromuscular junction between the nerve and muscles and eventually prevent nerve impulses from triggering muscle contractions. The main symptoms of myasthenia gravis are painless weakness of specific muscles. The weakness becomes progressively worse during physical activity and improves at rest. Several in vivo studies have reported the effectiveness of TKM treatment with curative intent in patients with this autoimmune synaptopathy [4]. In an animal study addressing the effect of acupuncture on the acetylcholine receptors at the neuromuscular junction, needles were inserted at the acupressure points Shousanli (LI10), Zusanli (ST36), Pishu (BL20), and Shenshu (BL23) once daily for 7 consecutive days.

The area and the integrated optical density of the immunoreactivity for the acetylcholine receptor at the neuromuscular junction of the phrenic nerve were significantly increased following acupuncture treatment [5].

Another study investigated the protective role of herbal medicine, Sizunzi decoction, in the neuromuscular junction. Intervention with Sizunzi decoction resulted in the findings of close to normal NMJ structure and significantly increased expression of the acetylcholine receptor and agrin [6].

Amyotrophic lateral sclerosis (ALS) is a disease characterized by abnormal function of the voluntary muscles. This malfunction results from death of the neurons which control voluntary muscles [7]. ALS is characterized by stiff muscles, muscle twitching, and gradually worsening weakness due to decreasing sizes of the muscles. The main mechanism of death of the motor neurons is the inflammatory process in the nervous system, which mainly involves mediators such as HLA-DR, tumor necrosis factor- (TNF-) alpha, interleukin- (IL-) 6, IL-beta, CD14, CD7, TLR4, iba-1, and glial fibrillary acidic protein (GFAP) positive astrocytes and CD11b positive microglial cells. Several studies have reported on the anti-inflammatory effect of TKM treatment [8].

Most of the studies have focused on bee venom pharmacoacupuncture at the Zusanli (ST36) acupoint. An animal study investigating the effect of bee venom pharmacoacupuncture on the central nervous system and muscle in an animal model of ALS showed that bee venom treatment at the Zusanli acupoint significantly enhanced motor function and decreased motor neuron death in the spinal cord compared to the control group. Furthermore, this treatment eliminated downstream signaling of inflammatory proteins, such as TLR4, in the spinal cord, and reduced levels of TNF-alpha and Bcl-2 expression in the ALS animal model [9].

In another similar animal study, bee venom treated animals reported decreased expression levels of microglia marker and phospho-p38 MAPK in the spinal cord and brainstem; moreover, treatment with bee venom improved motor activity in animals with symptomatic ALS [10].

Another study explored the effect of electroacupuncture treatment at the Zusanli acupoint. This study reported that ALS animal models treated with electroacupuncture showed a decrease in microglial cell activity and TNF-alpha expression in the spinal cord and brain stem. In addition, the treatment significantly improved motor activity compared to that in the control group and reduced neuronal cell loss in the ALS model [11].

In a study investigating the effect of an herbal medicine, Wen-Pi-Tang, on the ALS animal model showed that the Wen-Pi-Tang treatment inhibited neuronal loss in the lumbar segment of the spinal cord of mice. In addition, astrocytes and CD11b positive microglial cell in the spinal cord that increase prior to neuronal loss were significantly reduced in the Wen-Pi-Tang treated group [12].

Multiple sclerosis is a demyelinating disease with three main characteristics: plaque formation, inflammation, and destruction of the myelin sheaths of neurons. The insulating covers of nerve cells in the brain and spinal cord are damaged and this disrupts the ability of parts of the nervous system to communicate, resulting in muscle weakness and motor dysfunction [13]. Apart from demyelination, inflammation is the other main cause of the disease. The T cells gain entry into the brain via disruptions in the blood brain barrier. Further breakdown of the blood brain barrier causes a number of other damaging effects such as swelling, activation of macrophages, and increased activation of cytokines and other destructive proteins. Inflammation can reduce transmission of information between the neurons [14].

In a study investigating the effect of bee venom in suppression of motor neuron loss and microglial cell activation, bee venom was injected at the Zusanli (ST36) acupoint in the experimental group. As a result, the symptoms of clinical disorder, pathologic changes, inflammatory cell infiltration, demyelination in the central nervous system, and levels of TNF-alfa, and serum nitrates were decreased in the multiple sclerosis model [15].

Another study investigated whether Hyung bangpaedok-san (HBPDS), a traditional herbal medicine, has a beneficial effect in the multiple sclerosis model in rats. Onset-treatment with HBPDS alleviated neurological signs, reduced demyelination and the infiltration of microglia and macrophages, and reduced the mRNA expression of proinflammatory cytokines and chemokines in the spinal cord [16].

Guillain-Barre syndrome is a rapid-onset muscle weakness caused by immune system-mediated damage to the peripheral nerve system. The muscle weakness begins in the feet and hands. This often spreads to the arms and upper body with bilateral involvement. The cause and mechanism of this disease is unknown [17].

A study investigated the mechanism of electro acupuncture at the Shu-points of the five zang-organs for treatment of Guillain-Barre syndrome. In this study, electroacupuncture treatment increased the sciatic nerve movement conduction velocity and decreased the abnormal F wave incidence rate of the sciatic nerve in the Guillain-Barre syndrome model group [18].

Duchenne muscular dystrophy is a severe type of muscular dystrophy caused by X-linked recessive gene. The muscular weakness is associated with muscle wasting of the voluntary muscles. The hips, pelvic area, thighs, shoulders, and calves are the first to be affected; subsequently, muscle weakness occurs in the arms, neck, and other areas [19].

A study accessed the effects of herbal medicine on Duchenne muscular dystrophy. This study showed that the Liu-Wei-Di-Huang-Wan (LDW) and San-Lin-Pai-Tsu-San (SPS) can facilitate locomotor activity with the parameters of horizontal activity, total distance, number of movements, movement time, vertical activity, number of vertical movements, and vertical movement time [20].

Polymyositis is a chronic inflammation of muscles related to dermatomyositis and inclusion body myositis. The hallmark of this disease is weakness and loss of muscle mass in the proximal musculature, as well as flexion of the neck and torso [21].

A study investigated the therapeutic effects of Radix Bupleuri and Ramulus Cinnamomi on the polymyositis model. The herbal medicine treatment significantly increased expression of MyoD and myogenin and the function of myosin heavy chain and activated the Akt/mTOR pathway. In addition, the treatment suppressed the TNF-alpha activation [22].

Due to lack of understanding of the cause and mechanism of wilting disorder, allopathic treatment is generally used to relive the patients' symptoms of pain and weakness. We reviewed the in vitro studies focusing on diseases that can cause the symptoms of wilting disorder and reported our results. Our results indicated that TKM was effective in improving the electrophysiological status, histological and biochemical measurement values, and motor function value index, especially in animal models of myasthenia gravis, amyotrophic lateral sclerosis, and multiple sclerosis. However, there was lack of evidence for the TKM treatment effect in the Guillain-Barre syndrome, Duchenne muscular atrophy, and polymyositis models.

This study has some limitations. First, the use of limited search strategies returned a small number of studies. However, very few studies have been reported on the effect of TKM on wilting disease. Second, it was difficult to follow a strictly standardized method of analysis because of limited access to complete data from the studies. Future research is required to fully evaluate the effectiveness of TKM treatment in animal models.

## Figures and Tables

**Table 1:** Summary of articles on Traditional Korean Medicine treatment for diseases that can be main causes of wilting disorder.

**Author ** **(year)**	**Study design**	**Model**	**Sample size **	**Outcome measure**	**Intervention group**	**Control group**	**Results**
Hai-peng H (2016)	In vivo	Experimental autoimmune myasthenia gravis model	70 (Normal 10 + MG 20 + Acupuncture 20 + Drug 20)	(1) Immunoreactivity for AchR	A: AT (LI10, ST36, BL20, BL23, 30 min, once a day, 7 days)	B: MG w/o treatment C: Medicine	(1) Immunoreactivity for AchR at NMJ: AT > drug (p<0.01)

Wu H (2013)	In vivo	Experimental autoimmune myasthenia gravis model	60 (MG 20 + prednisone 20 + Sijunzi decoction 20)	(1) NMJ ultrastructure (2) Muscle cell mitochondria (3) ATP (4) Mitochondrial respiratory chain complexes	A: Sizunzi decoction (1 mg/kg, once a day, for 14 days)	B: Prednisone	(1) NMJ ultrastructure: control & prednisone (sparse, diffluent, absent) Sizunzi decoction (close to normal) (2) Gastrocnemius muscle mitochondrial count: Sizunzi decoction > prednisone > model (p<0.01) (3)-(4) Activities of gastrocnemius muscle mitochondrial respiratory chain: Sizunzi decoction > prednisone (Complexes I, III) (p<0.05)

Mudan C (2015)	In vivo	Amyotrophic lateral sclerosis model	60 (Normal 15 + ALS saline 15 + ALS BV 15 + ALS BV ST36 15)	(1) Footprint test (2) Motor Neuron cell death (3) TLR-4 & CD14 & TNF-*α*	A: BV (0.1*μ*g/g, ST36 or intraperitoneal, twice a week, 4 times)	B: Saline C: Riluzole	(1) Motor activity (Footprint test): BV ST36 > BV intraperitoneal > Saline (p<0.05) (2) Quantification of motor neurons in L4-5: BV ST36 > BV intraperitoneal > Saline (p<0.05) (3) TLR4 signaling related inflammatory protein, CD14, TNF-alpha↓ (p<0.05)

EJ Yang (2010)	In vivo	Amyotrophic lateral sclerosis model	45 (Saline 15 + BV 15 + Riluzole 15)	(1) Rotarod test (2) TNF-alpha expression (3) ERK 1/2 signaling, p-Akt	A: BV (0.1 *μ*g/g, ST36, twice a week, 4 times)	B: Saline C: Riluzole	(1) Motor activity (Rotarod test): BV > Riluzole > Saline (p<0.005) (2) TNF-alpha reduction: BV > Riluzole (p<0.001) (3) ERK1/2 signaling, p-Akt ↑

EJ Yang (2010)	In vivo	Amyotrophic lateral sclerosis model	28 (EAT 15 + Control 13)	(1) Rotarod test (2) Iba1, MAP2 protein, Neu N positive cells (3) TNF-alpha expression	A: EAT (1 mA, 2 Hz, 30 min, ST36, every two days, 28 days)	B: ALS w/o treatment	(1) Motor activity (Rotarod test): EAT > Control (p<0.001) (2) Iba-1 reduction & Neu N positive cells: EAT > Control (p<0.005) (3) TNF-alpha inhibition: EAT > Control (p<0.001)

Michiko S (2015)	In vivo	Amyotrophic lateral sclerosis model	60 (Model 15 + WPT100 15 + WPT200 15 + Riluzole 15)	(1) Rotarod test, wire hang test, grip strength test (2) Disease onset time (3) Number of motor neurons (4) Astrocytic & microglial cell activation	A: Wen-Pi-Tang (100 & 200 mg/kg, once a day, 14 days)	B: ALS w/o treatment C: Riluzole	(1) Motor function test: test - WPT200 > WPT100 > Riluzole after 10 wks (p<0.05) (2) Disease onset delay: WPT200 > Riluzole (p<0.01) (3) Number of motor neurons: WPT200 > WPT100 > Riluzole (p<0.05) (4) GFAP: WPT200 > Riluzole > WPT100 (p<0.05) CD11b-positive microglial cells: WPT200 > Riluzole (p<0.01)

Akbar K (2012)	In vivo	Multiple sclerosis model	30 (Saline 10 + BV 2mg 10 +BV 5mg 10)	(1) Rotarod test (2) Demyelination of CNS	A: BV (2 & 5 mg/kg, intraperitoneal, once a day, 20 days)	B: Saline	(1) Motor activity (Rotarod test): BV 5 mg, BV 2 mg > Saline (p<0.005) (2) Decreased demyelination of the CNS: BV 5 mg > BV 2 mg > Saline (p<0.005) (3) TNF-alpha reduction: BV 5 mg > BV 2 mg > Saline (p<0.005)

JH Choi (2015)	In vivo	Multiple sclerosis model	30 (Normal 10 + Control 10 + EAE & HBPDS 10)	(1) Behavioral score (2) Neurological impairment reduction (3) Demyelination (4) TNF-alpha, IL-1beta, MIP-1alpha, IL-6, RANTES, GAPDH,	A:Hyungbangpaedok-san (10-30 mg/kg, once a day, 21 days)	B: Multiple sclerosis w/o treatment	(1) Behavioral score: EAE + HBPDS30 > EAE (p<0.001) (2)-(3). Neurological impairment reduction & demyelination: EAE + HBPDS30 > EAE + HBPDS20 > EAE (p<0.001) (4) TNF-alpha ↓, IL-1beta ↓, MIP-1alpha ↓, IL-6 ↓, RANTES ↓, GAPDH ↓ (p<0.001)

Wang HF (2008)	In vivo	Guillain-Barre syndrome model	40 (model 20 + EAT 20 + immunoglobulin inj. 20)	(1) Sciatic nerve MCV (2) Abnormal F wave (3) Motor function test	A: EAT (1 mA, 5 Hz, LV3 LU9 SP3 KI3 HT7, 30 min, once a day, 14 days)	B: GB w/o treatment C: Immunoglobulin	(1) Sciatic nerve MCV: EAT > immunoglobulin > model (p<0.01) (2) F wave abnormal cases ↓ (3) BBB locomotor rating scale: EAT > immunoglobulin > model (p<0.01)

Chen SS (2001)	In vivo	Duchenne muscular dystrophy model	40 (Control 20 + Herbal medicine 20)	(1) Rotarod test (2) BBB locomotor rating scale & locomotor activity (3) Electrophysiological state	A: Liu-Wei-Di-Huang -Wan and San-Lin-Pai-Tsu-San 100 mg/kg, once a day, 90 days	B: DMD w/o treatment	(1), (2) Rotarod test / BBB locomotor rating scale: Herbal medicine group > Control, horizontal activity, total distance, vertical movement ↑ (p<0.001) (3) EMG: amplitude↑, duration↑

Chu X (2008)	In vivo	Polymyositis model	40 (Control 20 + Herbal medicine 20)	(1) MyoD, Myogenin, MHC (myosin heavy chain) (2) Akt / mTOR (3) TNF-alpha activation	A: Radix Bupleuri & Ramulus Cinnamomi (100 mg/kg, once a day, 14 days)	B: Polymyositis w/o treatment	(1) MyoD, Myogenin, MHC: Herbal medicine > Control (p<0.05) (2) Akt / mTOR activation↑ (3) TNF-alpha reduction ↑
